# Use of antenatal care services among extremely marginalized indigenous population (Chepang Community) of Nepal

**DOI:** 10.1371/journal.pgph.0004080

**Published:** 2025-03-13

**Authors:** Manita Bartaula, Manish Bartaula, Nishchal Devkota

**Affiliations:** 1 Department of Public Health, National Open College, Sanepa, Lalitpur, Nepal; 2 National Cardiac Center, Kathmandu, Nepal; Tribhuvan University Institute of Medicine, NEPAL

## Abstract

In Nepal, marginalized communities like the Chepang, who face socio-economic challenges, have limited access to healthcare services, resulting in low utilization of antenatal care (ANC) despite the availability of free maternal healthcare services. This study used a cross-sectional design using semi-structured questionnaires and in-person interviews to find out the factors influencing the use of ANC services among 130 women from Chepang (*an extremely marginalized ethnic tribe*) community in Rapti Municipality, Chitwan, Nepal. The collected data were entered in Epi-data and analyzed in SPSS ver. 16. Descriptive statistics were used to present the results that included the computation of frequency and percentages. Bi-variate logistic regression analysis followed by multi variable logistic regression analysis were used for predictor's analysis at 95% confidence interval with odds ratios. The utilization of ANC services was 76.9%, indicating that at least one visit was made. However, the utilization decreased to 43% for those who attended complete set of four or more visits. A majority of deliveries, comprising 62.3%, were assisted by individuals other than skilled health personnel. More than a half didn’t know about the government schemes of ANC services and around 13% were never respected on their decision by their husband to seek health care. Adjusted analyses indicated that heard of government schemes of ANC services use (AOR: 6.1, 95% CI: 2.30-16.23) and husband’s respect towards their spouse decisions to seek health care (AOR: 8.22, 95% CI: 1.65-40.82) remained significant factors positively influencing ANC services use, while other factors became insignificant after adjustment. These findings suggest that customized interventions focusing on increasing awareness of government schemes on ANC services and promoting respect towards women’s decisions could be effective in improving ANC services use among the extremely marginalized Chepang women of Nepal.

## Introduction

People who are shut out of mainstream society may be classified as socially, economically, educationally, or culturally marginalized, with examples including those excluded based on immigration status, physical ability, age, gender identity, sexual orientation, race/ethnicity, color, or language, all of which stem from unequal power relations among social groups [1]. Nepal’s traditional framework is dominated by Brahmin and Chhettri ethnic groups, with Hinduism prevalent in the Kathmandu Valley and hill regions, while marginalized groups like Rai, Limbu, Tamang, Magar, Gurung, and Terai struggle with limited government representation and resource allocation [2]. The Chepang, an indigenous group in Nepal, are classified as “highly marginalized” due to low literacy rates, limited land ownership, and scarce educational and employment opportunities. Despite transitioning from a nomadic lifestyle, they maintain their tribal identity through traditional knowledge and animistic practices, reflecting their cultural resilience [3]. Further, low-caste and marginalized women in Nepal face limited access to safe motherhood due to poor socio-economic status, poor knowledge, a lack of decision-making autonomy, and limited healthcare options [4].

Safe motherhood is essential for marginalized women, as it helps reduce maternal and child mortality, yet millions in developing nations continue to face preventable complications, with 800 women dying every minute from pregnancy-related issues, over 99% of these deaths occurring in underdeveloped regions, particularly South Asia and sub-Saharan Africa [5]. By 2030, the sustainable development goal (SDG) 3.1 targets reducing maternal deaths to fewer than 70 per 100,000 live births. This ambitious goal emphasizes the global commitment to improving maternal health outcomes and ensuring that all women, including those from marginalized communities, have access to safe and quality maternal care [6].

Maternal mortality, driven by hemorrhage, sepsis, and eclampsia, disproportionately impacts marginalized women. Access to quality antenatal care improves survival outcomes [7]. The World Health Organization advises having at least eight ANC visits throughout a pregnancy in order to lower maternal morbidity and death. These visits include health promotion, education about warning signs, preparing for childbirth, and helping locate resources for treating pregnancy-related issues [8,9]. Although, ANC recommends eight contacts for better outcomes, many countries, including Nepal, continue to follow the Focused Antenatal Care (FANC) model with only four visits [10]. The Chepang community, comprising 0.29% of Nepal’s population, holds substantial presence in Chitwan, where 42% of their population resides, totaling 35,637 individuals [11]. Despite their significant population, the Chepang community faces health challenges due to a low literacy rate of 23%, limited knowledge of safe motherhood and early marriage practices, and limited access to healthcare, with only 25% receiving basic services from health posts and 40% having access to primary healthcare facilities [12]. Additionally, a prior study found that pregnant Chepang women used healthcare services less frequently than non-Chepang women [13] likely due to limited access to healthcare resources and services within Chepang community, which may lead to lower utilization of health services. The findings from two separate studies reveal significant gaps in ANC utilization among Chepang women. In the first study, conducted in Chitwan, it was found that 67.2% of Chepang women had fewer than four ANC visits, compared to only 26.9% of non-Chepang women. This highlights a substantial disparity in healthcare utilization, with Chepang women facing persistent challenges in accessing adequate maternal healthcare. The second study, further emphasizes these gaps among Chepang women revealing that while some respondents attended the recommended number of antenatal visits, a significant portion attended very few or none at all [12,14]. The utilization of antenatal services remains particularly low, despite the availability of free maternal healthcare services among Chepang women [4]. This highlights the importance of understanding the current gaps and the challenges that Chepang women face in accessing maternal healthcare services. Understanding factors influencing safe motherhood practices among Chepang pregnant women in Chitwan District is crucial for reshaping program planning in Nepal, helping to improve the usage of services in the area [13]. Pregnant women’s use of antenatal services can be studied to find differences, highlight barriers and the bottlenecks, and provide ways to increase utilization [15,16]. Therefore, the objective of this study was to assess the use of ANC services and the factors associated with their use among Chepang women.

## Materials and methods

### Study design and setting

A quantitative cross-sectional analytical study was conducted to identify the factors associated with the use of ANC services. The research focused on Chepang women residing in the Rapti Municipality of Chitwan.

### Study participants and sampling technique

Women with children under the age of two years old were individually selected for the study. The data collection started from 14^th^ March to 19^th^ March, 2024. The Rapti municipality was purposively chosen from among one metropolitan city, five municipalities, and one rural municipality in Chitwan. Within the Rapti Municipality, which comprised a total of 13 wards, four wards were purposively selected for the study. For data collection, every health posts in wards 1, 2, 3, and 6 were visited, and permissions were obtained from the health post in-charge to access the register of ANC visits. Women of Chepang ethnicity who had children under two years old were listed down from the register of respective wards. Within these selected wards, all listed women having children under two years old, providing informed consent were included in the study. The exclusion criteria were female community health volunteers (FCHVs) & other women health personnel having under two years old children.

### Sample size

The sample size for this study was determined using Cochran’s formula, based on a 91.1% prevalence of women using ANC services [17]. With a 95% confidence interval (CI) and a 5% margin of error, the initial sample size was calculated to be 125. Complete enumeration was carried out in all the selected wards where 130 mothers of under two years children were obtained which were slightly more than the minimum sample required calculated in the current study.

### Study variables

Use of ANC services were assessed by considering the usage of pregnancy-related services and services obtained from a healthcare facility as dependent variable. The number of antenatal appointments throughout a pregnancy is known as the ANC. Complete use of ANC was defined as four or more ANC visits, whereas incomplete use was indicated by one, two, or three ANC visits. We considered the independent variables as predisposing and enabling factors, similar to those in Andersen’s model as used in a cross-sectional study done in Nepal [18]. Factors, including predisposing factors further divided into two subtopics, socio-demographic and knowledge on ANC services were among the independent variables. The assessment of knowledge on ANC services revolved around questions regarding familiarity with the initiation of an ANC checkup, awareness of the danger signs during ANC, and understanding the risks associated with delaying ANC visits. Knowledge scores ranged from zero to three, and the median value was used as the cutoff to classify knowledge level [19]. Participants scoring one or less were categorized as having poor knowledge, while those scoring above one was categorized as having good knowledge. Likewise, enabling factors, such as monthly family income, distance to the nearest health facility, respect women in decision making for seeking care by their husband/partner, heard of government schemes on ANC services use, assistance sought during delivery & access to roads were taken as independent variables. The age of women is a discrete variable categorized into four groups: less than 20, 21-25, 26-30 and 31 and above. The Government of Nepal’s Safe Motherhood Program offers financial incentives to encourage women to attend ANC and give birth in health facilities. Women receive transportation support and cash incentives based on their region: NPR 3,000 for those in the mountain region, NPR 2,000 in the hill region, and NPR 1,000 in the Terai region. Additionally, women who complete their full ANC visits are given NPR 800. Based on this initiative, participants were asked whether they were aware of the government scheme for ANC. Further, respect women in decision making for seeking care by their husbands/partners is labeled as follows: “Always” means the partner consistently supports her decision, “Sometimes” means the support is occasional or inconsistent, and “Never” means the partner does not respect or support her decision to seek care at all. Semi-structured questionnaires were used, combined with in-person interviewing procedures, to gather the data. Predisposing factors & enabling factors were aligned in the questions, and a questionnaire was created by rigorously reviewing the literature [13,18–23]. Experts in the area examined the questionnaire to ensure its validity.

#### Data collection procedure.

During data collection, home visits were conducted, and questionnaires were administered through in-person interviews with each mother in private settings, away from other family members. Participants were fully informed about the study’s purpose, and their consent were obtained before the interview, with assurance that their responses would remain confidential and used solely for research purposes. Sensitive topics were approached respectfully, and participants were given the option to skip any questions they felt uncomfortable answering. The interviewer emphasized that all responses would be kept private, shared only with the research team, and no identifying information would be disclosed in the final analysis.

#### Statistical analysis.

The data collected were entered into Epi-data 3.1, and IBM SPSS version 16 was used for analysis. To express the data, descriptive statistical methods such as percentage and frequency have been used. A chi-square test was performed at first to see the association of dependent variable with the independent variables (S1 Table). Bivariate logistic regression analysis was performed to examine the relationship between the dependent and independent variables, yielding the un-adjusted odds ratio (UOR) at a 95% CI & multi variable logistic regression analysis was performed further yielding adjusted odds ratio (AOR) at 95% CI.

### Ethical consideration

We obtained ethical approval (ref no.: 080/81/291) from the Nobel College Institutional Review Committee (Nobel-IRC) and received a letter of support (ref no.: 4354) from the Rapti Municipality Chairperson for data collection. Written informed consent was obtained from literate participants, while verbal consent was taken from those unable to read or write. As the data collector, the first author ensured that all consent procedures were properly followed. This approach was approved by the Nobel-IRC, ensuring compliance with ethical standards in obtaining and documenting consent. The study participants voluntarily participated, and their privacies were protected.

## Results

More than half (58.5%) were aged between 21 and 25 years, with the second largest group being 26.2% in the 26–30 age range. Further, 24.6% of women were illiterate, 50.8% had primary education, 18.5% had secondary education, and 6.2% had higher secondary education. Regarding family size, 69.2% of the women had up to two children, while 30.8% had more than two. Only 44.6% of the women heard about government scheme on ANC. Almost half (46.2%) exhibited poor knowledge, while 53.8% demonstrated good knowledge on ANC services. Moreover, merely 3.8% of the respondents had attended the ANC more than four times, and 23.1% had never gone for an ANC checkup (Table 1).

**Table 1:** Socio-demographic factors, knowledge of antenatal care, and utilization of antenatal care services among Chepang women.

Variables	Frequency (n=130)	Percentage (%)
**Age**		
Less than 20	12	9.2
21-25	76	58.5
26-30	34	26.2
31 and above	8	6.2
**Women’s education**		
Not able to read and write	32	24.6
Primary Education	66	50.8
Secondary Education	24	18.5
Higher Secondary Education	8	6.2
**Parity**		
Up to 2	90	69.2
More than 2	40	30.8
**Knowledge on ANC services**		
Poor Knowledge	60	46.2
Good Knowledge	70	53.8
**Heard of government Scheme of ANC services use**		
Yes	58	44.6
No	72	55.4
**Use of ANC services**		
One time	9	6.9
Two times	19	14.6
Three times	16	12.3
Four times	51	39.2
More than four times	5	3.8
Never	30	23.1

Regarding monthly family income, 88.5% of the respondent's families earned up to 20,000 Nepali rupees, while 15 families (11.5%) had incomes exceeding 20,000. For 33.1% of women, the distance to the nearest ANC facility was about 15 minutes; for 30.8%, it was about 30 minutes; and for 36.2%, it was more than 40 minutes. About two third women found the roads leading to the nearest medical facility to be convenient and secure. The husbands who respect the decisions that their wives make independently without their interference was 33.1%, but 13.8% never respected the decisions taken by women regarding health care seeking with around half women sometimes being respected of their decision. Skilled health professional provided assistance in 37.7% and 62.3% of childbirths involving other individual such as midwives, family members and traditional birth attendants (Table 2).

**Table 2:** Economic status, accessibility, and decision-making influences on ANC services use among Chepang women).

Variables	Frequency (n=130)	Percentage (%)
**Monthly Family Income**		
Up to 20000	115	88.5
Above 20000	15	11.5
**Distance to the nearest health facility**		
Around 15 minutes	43	33.1
Around 30 minutes	40	30.8
More than 40 minutes	47	36.2
**Access to road**		
Safe	87	66.9
Unsafe	43	33.1
**Respect women in decision making for seeking care by their husband**		
Always	43	33.1
Sometimes	69	53.1
Never	18	13.8
**Assistance sought during delivery by**		
Skilled health personnel	49	37.7
Other persons	81	62.3

To determine how the independent variables affected the outcome (*use of ANC*), both un-adjusted and adjusted analyses were performed yielding un-adjusted odds ratio (UOR) & adjusted Odds Ratio (AOR). Several significant relationships in the use of ANC were found in the un-adjusted analysis. For example, women with more than two children had a 3.13-fold higher UOR of the result than women with two or fewer children (95% CI: 1.37–7.16, p = 0.007). Additionally, compared to families with incomes up to 20,000, those with incomes beyond 20,000 showed an increase of 4.27 times (95% CI: 1.28–14.26, p = 0.01). The level of knowledge, i.e., good knowledge, had a 5.75 higher UOR than the poor level of knowledge (95% CI: 2.63–12.56, p = < 0.001). However, after adjusting for potential confounding variables, the associations slightly changed. For example, women who had deliveries assisted by skilled health personnel became non-significant after adjustment (AOR: 0.64, 95% CI: 0.213–1.932, p = 0.430). Likewise, the association with knowledge of government schemes remained significant but with a slightly reduced odds ratio (AOR: 6.11, 95% CI: 2.30–16.23, p = <0.001), while the association with husbands respecting wife's decisions also remained significant but with a slightly increased odds ratio (AOR: 8.22, 95% CI: 1.65–40.82, p = 0.010) (Table 3).

**Table 3:** Factors associated with the use of antenatal care services among Chepang women.

Variables	Unadjusted analysis		Adjusted analysis	
	UOR (95% CI)	p-value	AOR (95% CI)	p-value
**Parity**				
Up to 2	*Ref.*	0.007^*^	*Ref.*	0.369
More than 2	3.13(1.37-7.16)		1.72(0.52-5.69)	
**Monthly Family income**				
Up to 20000	*Ref.*	0.01^*^	*Ref.*	0.355
Above 20000	4.27(1.28-14.26)		2.11(0.43-10.39)	
**Knowledge on ANC services**				
Poor Knowledge	*Ref.*	<0.001^*^	*Ref.*	0.223
Good Knowledge	5.75(2.63-12.56)		1.89(0.678-5.30)	
**Assistance sought during delivery by**				
Skilled Health Personnel	5.20(2.413-11.21)	<0.001^*^	0.641(0.213-1.932)	0.430
Other Person	*Ref.*		*Ref.*	
**Heard of government schemes of ANC services use**				
Yes	10.87(4.71-24.66)	<0.001^*^	6.11(2.30-16.23)	<0.001^*^
No	*Ref.*		*Ref.*	
**Distance to the nearest health facility**				
Around 15 minutes	4.67(1.85-11.75)	0.001^*^	2.03(0.36-11.21)	0.41
Around 30 minutes	4.52(1.77-11.53)	0.002^*^	(2.12(0.44-10.08)	0.342
More than 40 minutes	*Ref.*		*Ref.*	
**Access to roads**				
Yes	3.70(1.625-8.43)	0.002^*^	1.10(0.23-5.21)	0.89
No	*Ref.*		*Ref.*	
**Respect women in decision making for seeking care by their husband/partner**				
Always	6.53(1.82-23.40)	0.004^*^	8.22(1.65-40.82)	0.010^*^
Sometimes	1.86(0.55-6.30)	0.315	3.82(0.84-17.46)	0.08
Never	*Ref.*		*Ref.*	

* p value statistically significant at 95% CI, Ref.: Reference.

## Discussion

In this study, the utilization of ANC visit was determined to be 76.9%, indicating that at least one visit was made. Also, the percentage of respondents in the current study who made completed (four or more) ANC visit was relatively low, standing at 43%, while 23.1% never visited for ANC checkups. A study in urban privileged setting of Baglung Municipality, Nepal, reported 99.4% ANC attendance [22], contrasting sharply with our findings. In spite of having equal entitlements to ANC services, marginalized groups in Nepal consistently under-utilize government services compared to more privileged women, as comparisons reveal. Gaps in ANC utilization among marginalized women are due to limited awareness of its importance and restricted access to healthcare, often linked to reduced autonomy, which further is supported by the present study, where, heard of government scheme on ANC services use among Chepang women & respect women in decision making for seeking care by their husband were found as significant predictors for ANC services use. A study from Nepal further highlights that educating women about ANC can help bridge these gaps [12] and aim for higher quality health services [16]. These findings highlight critical gaps and challenges in maternal healthcare access and utilization. The low rate of skilled health personnel assisting in pregnancy & childbirth indicates a concerning deficiency in accessing essential maternal health services, potentially risking the well-being of mothers and newborns. Moreover, the low attendance rates at ANC appointments indicate barriers to accessing prenatal care, which is vital for ensuring a healthy pregnancy and reducing maternal and neonatal complications. Another similar finding from the study conducted within the Chepang community, where 39% of respondents did not attend any antenatal clinics [12], further emphasize the challenges faced by marginalized populations in accessing essential maternal healthcare services. Present study focuses on the Chepang women, examining cultural and knowledge barriers that affect ANC use in rural, underserved populations. While few studies address similar factors [4,7,21], our research highlights key determinants namely lack of knowledge on government scheme on ANC services use and husband's respect for healthcare decisions that influence ANC uptake among Chepang women. The Chepang-specific approach provides a deeper understanding of the unique barriers to maternal healthcare access, setting it apart from existing research and offering insights to improve healthcare access and outcomes particularly for Chepang community.

In the present study, only 37.7% of births were assisted by skilled health personnel, compared to 81.7% in Benin, Africa [24]. Although both regions face similar challenges, Benin has made remarkable progress, showing a clear gap in access to maternal healthcare in marginalized women of Nepal. Research conducted in Chitwan among non-marginalized women showed that non-health professionals assisted in a relatively small proportion of deliveries [16]. Furthermore, only about 13% of pregnancy complications among Chepang women were handled by health workers [12]. More than two third delivery in the Chepang community occurred at home as reported in one of the studies [4], highlighting the severe lack of healthcare access. These findings emphasize the disproportionate burden faced by Chepang women compared to their non-marginalized counterparts in accessing maternal healthcare. Our study found that more than half (53.8%) of the women had a good knowledge of ANC services, which is in line with findings from similar studies in Karnataka, India [25] and among Chepang women in Nepal [12]. However, this also highlights a significant gap, as nearly half of the women still lack adequate knowledge about ANC services. This further highlights the challenges that marginalized women face in accessing essential maternal health information and services. For example, over half (55.4%) of Chepang women were unaware of government healthcare schemes available for ANC, pointing to a significant gap in knowledge and potential barriers to accessing these services. This is supported by another study showing that only about one in five Chepang women took advantage of the delivery incentives offered by the Government of Nepal, emphasizing the continuing difficulties in reaching and engaging these communities with vital health programs [4].

The current study reveals a significant disparity in healthcare access, with 36.2% of women facing lengthy travel times of over 40 minutes to reach the nearest healthcare facility. In contrast, women in the more privileged area of Chitwan district experience much shorter travel times, with 89.7% reporting it takes about 30 minutes to reach a healthcare facility [26]. These differences highlight systemic inequalities in healthcare access, with marginalized women particularly affected by barriers such as limited awareness and geographic distance, which can lead to delays in seeking and receiving care. However, it is worth noting that over half of the women in the study (66.9%) report that the roads to healthcare facilities are safe, which may encourage them to seek medical assistance when necessary. About 13.8% of husbands show disinterest in their wives healthcare decisions, suggesting communication challenges. However, 53.1% sometimes support their wives choices, and 33.1% consistently respect them, highlighting some positive aspects of women's decision-making autonomy in the present study. Child marriage in the Chepang community limits education, health, and decision-making power for girls, reinforcing poverty and inequality [27]. Heard of government schemes for ANC services use (UOR: 10.87, 95% CI: 4.71-24.66 and AOR: 6.11, 95% CI: 2.30-16.23) and husbands always respecting women in decision making for seeking care (UOR: 6.53, 95% CI: 1.82-23.40 and AOR: 8.22, 95% CI: 1.65-40.82) were significant predictors of whether Chepang women used ANC services. These findings are unique to Chepang women and consistent with research from other marginalized communities in Nepal [7,21], emphasizing how important it is for women to feel supported in their healthcare choices. When husbands respect and actively back their wives decisions about their health, it creates a positive and empowering environment that encourages women to seek the care they need [7]. While parity, monthly family income, knowledge on ANC services, delivery assistance by skilled health personnel, proximity to health facilities, and access to roads turned insignificant in the adjusted analysis, their importance should not be underestimated. These factors still have the potential to influence maternal health, but their effects may be less direct or may interact with other determinants, such as access to care, social support, and education, which could further explain the low use of ANC services among Chepang women, in addition to the identified predictors in the study. Addressing these factors, even if not statistically significant in every model, remains critical to creating an environment that supports maternal health at multiple levels.

### Limitation of the study

The Chepang women with children under two years were unevenly distributed across the wards of Chitwan district however, due to the limited sample size of this demographic group, there may be wider confidence intervals for certain variables after applying logistic regression model. Additionally, the subjective nature of respondents’ answers could potentially influence result accuracy, warranting cautious interpretation.

## Conclusion

The study reveals that the use of ANC services is lower among Chepang women, as more than half did not receive the recommended four or more ANC visits, as per the Nepal government's guidelines for minimum antenatal care during pregnancy. The factors for the non-use of the services are poor access in the information related to ANC schemes of government and the women's autonomy. Women are more likely to use these services if they are aware of government schemes of ANC services and husbands who accept their decisions. Policy implications should focus on increasing awareness of ANC services and government schemes, especially through community-based education initiatives, such as female community health volunteers, outreach programs, and digital platforms. Policies should prioritize empowering mothers by improving their autonomy in healthcare decisions, including couple counseling and gender equity dialogues to involve husbands. Additionally, customized interventions are needed to improve accessibility to ANC services, particularly in remote areas, and address cultural and gender norms that hinder women's health decision-making, ultimately improving maternal health outcomes for Chepang women.

## Supporting information

S1 Table Chi square table of association between use of ANC and independent variables. (DOCX)

S1 STROBE Checklist STROBE statement. (DOCX)

S1 Checklist Inclusivity in global research. (DOCX)
