# Supplementary material for: Use of antenatal care services among extremely marginalized indigenous population (Chepang Community) of Nepal
Source: PLOS Glob Public Health. 2025 Mar 13;5(3):e0004080. doi: 10.1371/journal.pgph.0004080 (PMC11906166; doi:10.1371/journal.pgph.0004080)
Supplement: S1 Table — (DOCX) [file pgph.0004080.s001.docx]

**S1 Table: Association of Use of ANC with independent variables**

| Variables | | Utilize | | χ2 | p-value |
| --- | --- | --- | --- | --- | --- |
|  |  | **Incomplete** | **Complete** |  |  |
| Parity | Up to 2 | 44 (59.5%) | 46 (82.1%) | 7.700 | **0.006*** |
|  | More than 2 | 30 (40.5%) | 10 (17.9%) |  |  |
| Family income | Up to 20,000 | 70(94.6%) | 45(80.4%) | 6.331 | **0.012*** |
|  | Above 20, 000 | 4(5.4%) | 11(19.6%) |  |  |
| Knowledge on ANC | Poor Knowledge | 47 (63.5%) | 13 (23.2%) | 20.831 | **< 0.001*** |
|  | Good Knowledge | 27(36.5%) | 43 (76.8%) |  |  |
| Assisted for delivery | Skilled Health Personnel | 16(27.9%) | 33  (21.1%) | 18.89 | **< 0.001*** |
|  | Others | 58(46.1%) | 23(34.9%) |  |  |
| Knowledge on government schemes | Yes | 16 (21.6%) | 42(75.0%) | 36.756 | **< 0.01** |
|  | No | 58(78.4%) | 14(25.0%) |  |  |
| Nearest health facilities | Around 15 minutes | 19 (25.7%) | 24 (42.9%) | 14.273 | **< 0.001*** |
|  | Around 30 minutes | 18 (24.3%) | 22 (39.3%) |  |  |
|  | More than 40 minutes | 37 (50%) | 10 (17.9%) |  |  |
| Easily accessible and safe roads | Yes | 41(55.4%) | 46 (82.1%) | 10.295 | **0.001*** |
|  | No | 33 (44.6%) | 10 (17.9%) |  |  |
| Husband respect decisions you made | Always | 15 (20.3%) | 28 (50.0%) | 13.646 | **0.001*** |
|  | Sometimes | 45 (60.8%) | 24 (42.9%) |  |  |
|  | Never | 14 (18.9%) | 4 (7.1%) |  |  |

**p value less than 0.05 is statistically significant at 95% confidence interval (CI)*
